# Striatal Metabolomic Profiling Links Brazilian Green Propolis to Suberic Acid Modulation and Nigrostriatal Neuroprotection in a Rat Model of Parkinson’s Disease

**DOI:** 10.3390/molecules31111791

**Published:** 2026-05-23

**Authors:** Kételin Vitória Matias, Mario Augusto Izidoro, Fernando Barbosa, Bruno Alves Rocha, Victor Silva da Fonsêca, Fulvio Alexandre Scorza, Frederick Wasinski, Valeria de Cassia Gonçalves, Rozana Mesquita Ciconelli, Andresa Aparecida Berretta, Josef Finsterer, Carla Alessandra Scorza

**Affiliations:** 1 Disciplina de Neurociência, Departamento de Neurologia e Neurocirurgia, Universidade Federal de São Paulo (UNIFESP), São Paulo 04039-032, SP, Brazil; ketelin.matias@unifesp.br (K.V.M.); scorza@unifesp.br (F.A.S.); f.wasinski@unifesp.br (F.W.); vaal.cassia@gmail.com (V.d.C.G.); 2 Laboratório de Espectrometria de Massas, Hospital São Paulo (HSP), São Paulo 04024-002, SP, Brazil; mario.izidoro77@gmail.com; 3 Analytical and System Toxicology Laboratory, Department of Clinical Analyses, Toxicology and Food Sciences, School of Pharmaceutical Sciences of Ribeirao Preto, University of Sao Paulo, Ribeirão Preto 04021-001, SP, Brazil; fbarbosa@fcfrp.usp.br; 4 Institute of Chemistry, Federal University of Alfenas, Alfenas 37130-001, MG, Brazil; 5 Instituto Israelita de Ensino e Pesquisa, Faculdade Israelita de Ciências da Saúde Albert Einstein, Hospital Israelita Albert Einstein, São Paulo 05652-000, SP, Brazil; 6 Departamento de Pesquisa da BP, a Beneficência Portuguesa de São Paulo, São Paulo 01323-001, SP, Brazil; rozana.ciconelli@bp.org.br; 7 Laboratory of Research, Development and Innovation, Apis Flora Indl, Coml, Ltda., São Paulo 14020-670, SP, Brazil; andresa.berretta@apisflora.com.br; 8 Neurology and Neurophysiology Center, 1180 Vienna, Austria; fifigs1@yahoo.de

**Keywords:** Parkinson’s disease, propolis, natural products, suberic acid, peroxisomal dysfunction, neuroprotection, metabolism, oxidative stress, mitochondrial dysfunction

## Abstract

Parkinson’s disease (PD) is characterized by progressive nigrostriatal degeneration and striatal dysfunction, yet its metabolic remodeling remains incompletely defined. Here, untargeted GC–MS metabolomics was used to investigate the effects of standardized Brazilian green propolis on the striatal metabolic profile in the 6-hydroxydopamine (6-OHDA) rat model. Discriminant metabolites, including suberic acid, gluconic acid, heptadecane, and tartaric acid, distinguished experimental groups, capturing key features of the metabolic response to dopaminergic injury and treatment. Suberic acid emerged as a prominently modulated metabolite, potentially linked to alterations in lipid catabolism associated with mitochondrial–peroxisomal pathways. Propolis treatment attenuated the elevation of suberic acid, accompanied by a reduction in gluconic acid levels, suggesting a metabolic profile linked to pathways involved in redox balance and glucose handling. Given previous reports identifying heptadecane as a hydrocarbon constituent of volatile propolis fractions, complementary GC-Q-TOF analyses demonstrated that heptadecane was absent from the administered extract, despite its consistent association with propolis-treated groups. Metabolic changes were accompanied by attenuation of nigrostriatal dopaminergic neurodegeneration and improved motor performance. Together, these findings delineate a striatal metabolic signature associated with Brazilian green propolis and identify suberic acid as a key metabolite linked to neuroprotection in experimental Parkinsonism.

## 1. Introduction

Parkinson’s disease (PD) is a multifactorial neurodegenerative disorder characterized by the progressive loss of dopaminergic neurons in the substantia nigra pars compacta and subsequent depletion of striatal nerve terminals. As the main recipient of nigral dopaminergic input, the striatum plays a pivotal role in motor control while integrating cognitive, emotional, and reward-related circuits [1,2,3,4]. Despite representing a central hub in which multiple molecular and signaling pathways converge, its comprehensive metabolic remodeling in PD remains insufficiently explored [5,6]. Current pharmacotherapies for PD, primarily based on dopamine replacement, provide only symptomatic relief and fail to halt neurodegeneration or adequately address the wide spectrum of non-motor manifestations that impair quality of life [7,8]. These limitations highlight the need for a deeper understanding of the molecular and metabolic determinants underlying striatal dysfunction, with the aim of identifying novel therapeutic strategies. In this context, untargeted metabolomics has emerged as a powerful and unbiased approach to capture disease-related metabolic changes and to assess their modulation by biologically relevant interventions.

The 6-hydroxydopamine (6-OHDA) model reflects key neuropathological hallmarks of PD, such as mitochondrial dysfunction, oxidative stress, and neuroinflammation, through selective nigrostriatal dopaminergic damage, and provides a robust platform for investigating the metabolic correlates of degeneration and neuroprotection [9,10,11].

Brazilian green propolis, a resinous product derived from plant exudates collected by honeybees, has attracted increasing attention as a multifunctional natural compound with translational potential. Enriched with flavonoids, terpenes, and phenolic acids, it exerts broad modulatory effects on oxidative, inflammatory, immunometabolic, and mitochondrial pathways [12,13,14]. Our previous findings showed that standardized Brazilian green propolis attenuates striatal degeneration in rats with 6-OHDA lesions [15,16], although the underlying metabolic mechanisms remain largely undefined.

The aim of this study was to profile striatal metabolic alterations induced by 6-OHDA and their modulation by Brazilian green propolis using untargeted GC–MS-based metabolomics. This approach provides insights into metabolic remodeling in PD and elucidates the biochemical basis of propolis-mediated metabolic modulation.

## 2. Results

### 2.1. Brazilian Green Propolis Mitigates 6-OHDA-Induced Loss of Nigral Dopaminergic Neurons

**Figure 1:**
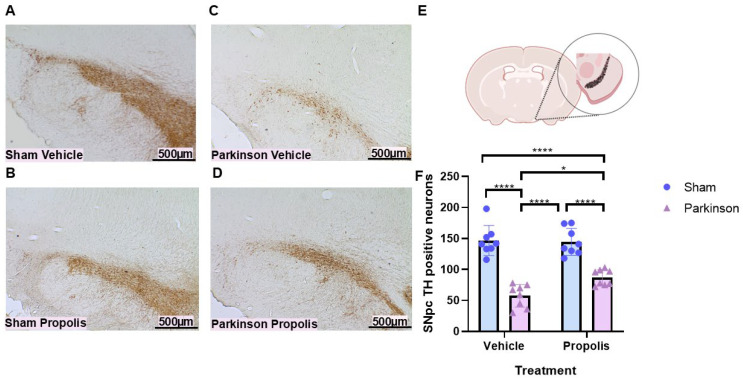
Tyrosine hydroxylase (TH) immunostaining of dopaminergic neurons in the substantia nigra pars compacta (SNpc). Representative photomicrographs show dense TH-positive neurons in (**A**) Sham + Vehicle and (**B**) Sham + Propolis, a marked reduction in (**C**) Parkinson + Vehicle, and partial preservation in (**D**) Parkinson + Propolis (*n* = 8 per group). (**E**) Diagram showing the anatomical localization of the SNpc. (**F**) Quantitative analysis reveals significantly higher TH-positive neuronal counts in Parkinson + Propolis group compared with Parkinson + Vehicle group. Data are expressed as mean ± SEM. **** *p* < 0.0001, * *p* < 0.05.

Tyrosine hydroxylase (TH)-positive immunoreactivity was quantified in the substantia nigra pars compacta (SNpc) across experimental groups (Figure 1A–D), with anatomical localization shown in Figure 1E and neuronal counts summarized in Figure 1F. Sham + Vehicle and Sham + Propolis groups exhibited comparable numbers of TH-positive neurons, indicating that propolis administration alone did not affect basal dopaminergic integrity. In contrast, Parkinson + Vehicle animals displayed a 61% reduction in TH-positive neurons relative to Sham controls, confirming extensive dopaminergic degeneration. Propolis treatment attenuated this loss, preserving approximately 59% of TH-positive neurons, representing a 33% improvement relative to Parkinson + Vehicle. These findings indicate that standardized Brazilian green propolis mitigates dopaminergic cell loss in the SNpc under 6-OHDA-induced neurotoxicity.

### 2.2. Brazilian Green Propolis Improves Motor Performance in the Rotarod Test

**Figure 2:**
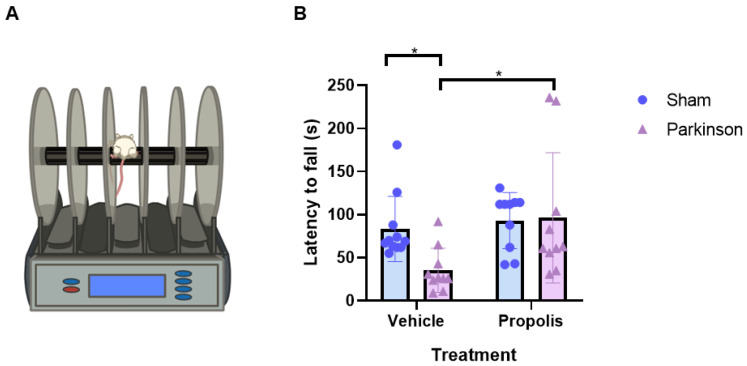
Brazilian green propolis improves motor performance in the rotarod test in the 6-OHDA model of Parkinson’s disease. (**A**) Schematic representation of the rotarod apparatus used to assess motor coordination and balance. (**B**) Latency to fall (s) across experimental groups: Sham + Vehicle, Sham + Propolis, Parkinson + Vehicle, and Parkinson + Propolis. Parkinson + Vehicle animals exhibited significantly reduced latency to fall compared with Sham + Vehicle, indicating motor impairment induced by 6-OHDA lesion. Propolis treatment significantly increased latency in Parkinsonian animals compared with Parkinson + Vehicle, restoring performance to levels comparable to Sham groups. No significant differences were observed between Sham groups or between Sham + Vehicle and Parkinson + Propolis. Data are presented as mean ± SD, with individual data points representing single animals. Statistical analysis was performed using a generalized linear model (gamma distribution with log link), followed by Mann–Whitney U test with Bonferroni correction. * *p* < 0.05.

Rotarod performance (Figure 2) revealed clear group-dependent differences consistent with motor impairment induced by dopaminergic lesion and its modulation by treatment. As illustrated in Figure 2A, the rotarod apparatus was used to assess motor coordination and balance. Quantitative analysis of latency to fall (Figure 2B) showed that Parkinson + Vehicle animals exhibited reduced performance compared with control groups. Group means (±SD) were as follows: Sham + Vehicle = 83.4 ± 37.7 s; Sham + Propolis = 93.0 ± 32.5 s; Parkinson + Vehicle = 35.4 ± 25.4 s; and Parkinson + Propolis = 96.2 ± 75.6 s. Analysis using a generalized linear model (gamma distribution with log link) revealed a significant interaction between lesion and treatment (F(1, 37) = 6.21; *p* = 0.017), indicating that the effect of propolis differed according to experimental condition. Parkinson + Vehicle animals exhibited a marked reduction in latency to fall compared with Sham + Vehicle, confirming impaired motor performance following 6-OHDA lesion. In contrast, propolis treatment significantly increased latency in Parkinsonian animals, restoring performance to levels comparable to Sham groups. No significant effect of propolis was observed in Sham animals (*p* = 0.679). Post hoc pairwise comparisons (Mann–Whitney U test with Bonferroni correction) supported these findings. Parkinson + Vehicle animals showed significantly lower latency than Sham + Vehicle (U = 97.0; padj = 0.014; r = 0.64), whereas Parkinson + Propolis animals exhibited significantly higher latency than Parkinson + Vehicle (U = 15.5; padj = 0.040; r = 0.58). No significant differences were detected between Sham + Vehicle and Parkinson + Propolis (padj = 1.000; r = 0.14) or between Sham groups (padj = 1.000; r = 0.15). Together, these results indicate that standardized Brazilian green propolis attenuates motor deficits induced by 6-OHDA without affecting baseline motor performance.

### 2.3. Protection of Striatal Dopaminergic Terminals by Brazilian Green Propolis

**Figure 3:**
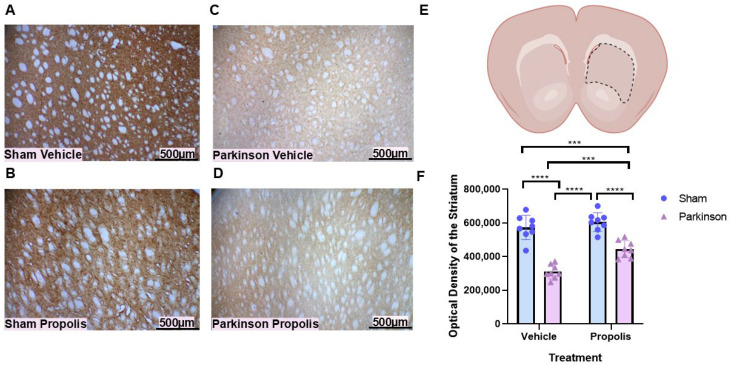
Standardized Brazilian green propolis preserves striatal dopaminergic fibers in the 6-OHDA model of Parkinson’s disease. (**A**) Sham + Vehicle. (**B**) Sham + Propolis. (**C**) Parkinson + Vehicle. (**D**) Parkinson + Propolis. Representative immunohistochemical staining of the striatum (scale bar, 500 µm). (**E**) Schematic representation of the analyzed striatal region, delineated by the dashed line. (**F**) Quantitative analysis of optical density revealed a pronounced reduction in TH immunoreactivity in Parkinson + Vehicle compared with Sham + Vehicle. Propolis treatment significantly attenuated this loss, increasing striatal fiber density in Parkinson + Propolis relative to Parkinson + Vehicle. Data are presented as mean ± SEM (*n* = 8 per group). Two-way ANOVA followed by Tukey’s post hoc test. *** *p* < 0.001, **** *p* < 0.0001.

Immunohistochemical analysis revealed pronounced group-dependent differences in striatal dopaminergic fiber density, as shown in Figure 3. Sham animals (A, B) exhibited dense, well-organized fibers with intense and homogeneous tyrosine hydroxylase (TH) immunoreactivity, confirming preserved striatal integrity. The Sham + Propolis group (B) closely resembled Sham + Vehicle (A), indicating that propolis administration did not alter basal dopaminergic architecture. In contrast, Parkinson + Vehicle animals (C) displayed a profound loss of TH-positive fibers, characterized by sparse, fragmented, and disorganized staining, consistent with 6-OHDA-induced denervation. Propolis treatment partially preserved striatal dopaminergic fibers in the Parkinson + Propolis group (D), which exhibited greater fiber density and more robust staining compared with Parkinson + Vehicle, although not fully restored to Sham levels. Quantitative optical density analysis (F) corroborated these observations, showing a significant reduction in the Parkinson + Vehicle group relative to Sham + Vehicle (**** *p* < 0.0001). Propolis treatment significantly increased optical density in Parkinsonian animals (*** *p* < 0.001 vs. Parkinson + Vehicle), supporting a neuroprotective effect. No significant differences were detected between Sham groups, reinforcing that propolis exerts protective effects selectively under neurotoxic conditions.

### 2.4. Metabolomic Landscape Modulated by Brazilian Green Propolis in the 6-OHDA Model of Parkinson’s Disease

**Figure 4:**
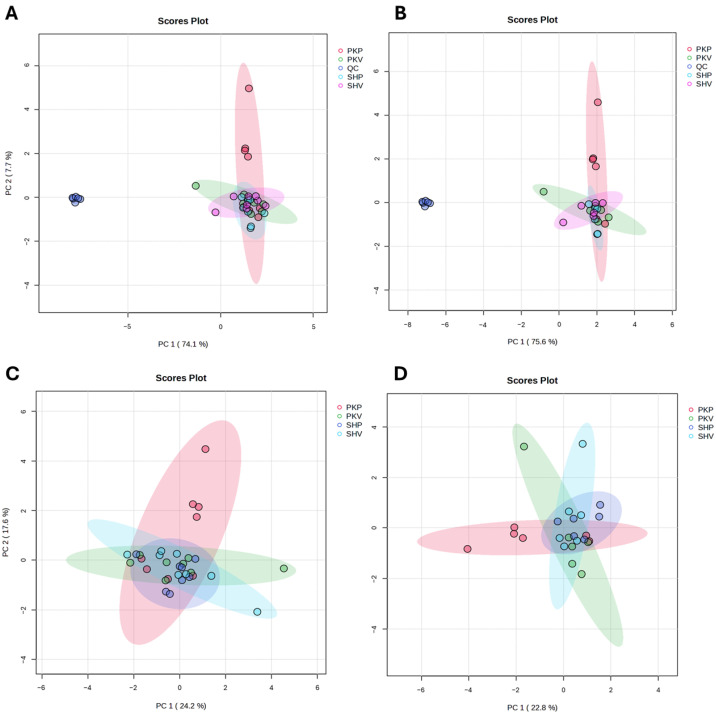
Principal component analysis (PCA) score plots showing the distribution of experimental groups before and after outlier removal and quality control filtering. (**A**) PCA including all samples before outlier removal. (**B**) PCA after removal of identified outliers. (**C**) PCA before outlier removal, excluding quality control (QC) samples. (**D**) PCA after removal of control samples and excluding QC samples. The statistical preprocessing applied included median normalization, log transformation, and Pareto scaling. Ellipses represent the confidence regions for each experimental group (PKP, PKV, SHP, SHV, and QC when applicable). The percentages on the axes indicate the variance explained by the first (PC1) and second (PC2) principal components.

Untargeted GC–MS metabolomic profiling of striatal tissue revealed marked metabolic alterations associated with dopaminergic lesion and its modulation by treatment with standardized Brazilian green propolis. Principal component analysis (PCA) was initially performed to explore the global structure of the metabolomic dataset (Figure 4). As expected, quality control (QC) samples clustered tightly in the multivariate space, supporting the analytical robustness, reproducibility, and instrumental stability of the GC–MS platform throughout the analytical sequence. Initial unsupervised PCA models indicated only subtle separation among experimental groups (Figure 4A). Notably, samples from the PKP group exhibited two distinct clustering tendencies, suggesting potential intra-group heterogeneity.

To further investigate whether these patterns reflected biological variability or the presence of anomalous samples, an additional outlier detection step was performed using a Random Forest-based approach. Random Forest analysis was applied to evaluate sample similarity using proximity measures generated during the ensemble classification procedure. From these proximity matrices, outlier scores were calculated based on the inverse of the average proximity of each sample to other samples belonging to the same class. Samples exhibiting substantially lower within-class proximity values were considered potential outliers. This approach enables the identification of samples that deviate from the overall structure of their respective experimental group in a data-driven and non-parametric manner, making it particularly suitable for high-dimensional metabolomic datasets. The Random Forest outlier detection identified two samples displaying consistently high outlier scores relative to their respective groups. These samples showed markedly reduced similarity to other biological replicates within the same experimental condition, indicating that their metabolomic profiles did not represent the typical metabolic phenotype of the group. Based on this criterion, these samples were excluded from subsequent multivariate analyses. Following the removal of these outliers, the dataset was re-normalized, and new PCA and PLS models were generated. The refined PCA model showed improved group organization while preserving the biological variability inherent to in vivo experimental designs (Figure 4B–D).

**Figure 5:**
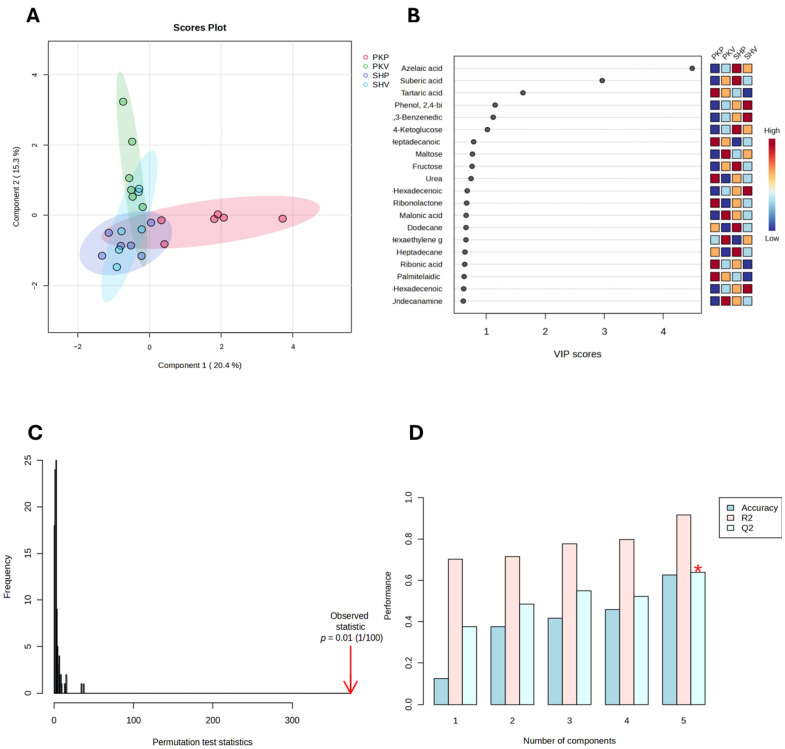
Multivariate analysis of metabolomic profiles among experimental groups (PKP, PKV, SHP, and SHV). (**A**) PLS-DA score plot showing the separation of groups based on Component 1 (20.4%) and Component 2 (15.3%), with ellipses representing the confidence intervals for each group. (**B**) Variable Importance in Projection (VIP) scores identifying the metabolites that most contributed to group discrimination; the heatmap on the right represents the relative abundance of these metabolites across groups (red = high abundance, blue = low abundance). (**C**) Permutation test (*n* = 100) used to assess the robustness of the PLS-DA model, indicating statistical significance (*p* = 0.01). (**D**) Model performance metrics showing Accuracy, R^2^, and Q^2^ values according to the number of components used in the model, with the optimal model indicated by the highest predictive performance. An asterisk (*) indicates the optimal number of components selected for the final PLS-DA model based on predictive performance.

Supervised multivariate modeling using partial least squares discriminant analysis (PLS-DA) further characterized metabolic differences among the experimental groups (Figure 5A). Model validation demonstrated improved performance after the removal of the identified outliers. Cross-validation parameters revealed substantial improvement in classification metrics, with accuracy increasing from 0.23 to 0.63, R^2^ from 0.67 to 0.90, and Q^2^ from 0.35 to 0.65 after outlier exclusion (Table 1). Permutation testing (*n* = 100) confirmed the statistical robustness of the final model (*p* = 0.01), indicating that the observed class separation was unlikely to be the result of model overfitting (Figure 5C). Variable Importance in Projection (VIP) analysis identified a subset of metabolites contributing most strongly to group discrimination (Figure 5B). These metabolites were subsequently evaluated using univariate statistical analysis.

**Figure 6:**
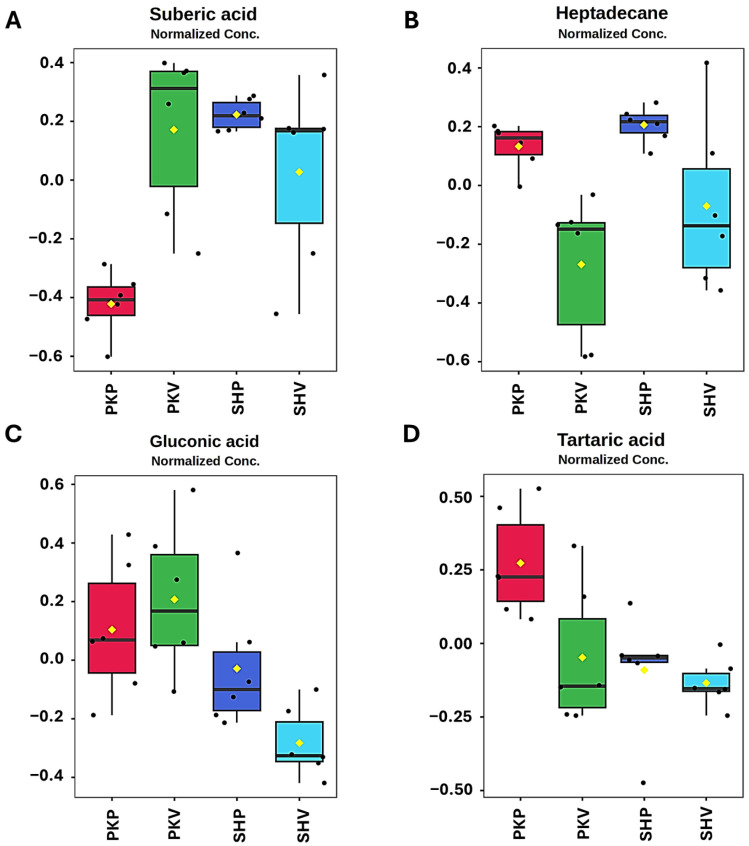
Differential striatal metabolites associated with Parkinsonian lesion and modulation by Brazilian green propolis. Boxplots illustrate the relative abundance of the four metabolites identified as significantly altered among experimental groups following one-way ANOVA with Tukey’s post hoc test and FDR correction: (**A**) suberic acid, (**B**) heptadecane, (**C**) tartaric acid, and (**D**) gluconic acid. Data were median-normalized, log-transformed, and Pareto-scaled prior to statistical analysis. Yellow diamonds indicate group means, and black dots represent individual data points. Significant group effects were observed for suberic acid (F(3, n) = 10.862; *p* = 0.00018; FDR = 0.0083), heptadecane (F(3, n) = 7.1186; *p* = 0.0019; FDR = 0.0426), tartaric acid (F(3, n) = 6.020; *p* = 0.0043; FDR = 0.0498), and gluconic acid (F(3, n) = 5.9485; *p* = 0.0045; FDR = 0.0498). Group abbreviations: SHV = Sham + Vehicle; SHP = Sham + Propolis; PKV = Parkinson + Vehicle; PKP = Parkinson + Propolis.

Complementary one-way ANOVA followed by Tukey’s post hoc test identified four metabolites showing significant group-dependent alterations (Table 2; Figure 6). The experimental groups were defined as SHV (Sham + Vehicle), SHP (Sham + Propolis), PKV (Parkinson + Vehicle), and PKP (Parkinson + Propolis). Suberic acid exhibited the strongest statistical effect among the detected metabolites (F(3, n) = 10.86; *p* = 0.000189; FDR = 0.0083). Tukey’s post hoc comparisons indicated significant differences between PKP and all other groups (PKV, SHP, and SHV). Heptadecane also differed significantly among groups (F(3, n) = 7.12; *p* = 0.00194; FDR = 0.043), with pairwise differences observed between PKV vs. PKP and SHP vs. PKV. Tartaric acid levels showed a significant group effect (F(3, n) = 6.02; *p* = 0.00428; FDR = 0.0498), driven primarily by higher levels in PKP relative to all other experimental groups. Finally, gluconic acid exhibited a significant group effect (F(3, n) = 5.95; *p* = 0.00453; FDR = 0.0498), with SHV differing significantly from both PKP and PKV.

### 2.5. Volatile Profile of Green Propolis

The GC-Q-TOF analysis of the essential oil obtained by hydrodistillation of raw Brazilian green propolis revealed a volatile profile predominantly composed of sesquiterpenes, oxygenated sesquiterpenes, hydrocarbons, and phenylpropanoids, consistent with previous reports on the chemical composition of green propolis derived from *Baccharis dracunculifolia* [17]. In contrast, the hexane extract obtained by liquid–liquid extraction of the propolis gavage formulation exhibited a considerably reduced number of volatile constituents. This observation is expected, as the preparation of the standardized propolis extract involves concentration and drying steps that likely result in the loss of the most volatile compounds during processing. Consequently, the LLE fraction was predominantly enriched in semi-volatile apolar constituents, including components derived from the castor oil vehicle.

#### Targeted Investigation of Heptadecane

The primary objective of this analysis was to confirm or rule out the presence of heptadecane (C_17_H_36_; *m*/*z* 240.2817) in the propolis samples. First, the total ion chromatograms acquired at 70 eV were subjected to automated spectral deconvolution (SureMass algorithm) followed by library search against the NIST 23 database. Heptadecane was not identified among the annotated compounds in either the raw propolis or the gavage formulation. Second, a targeted screening was performed by extracting the ion chromatogram (EIC) at the exact mass of the heptadecane molecular ion (*m*/*z* 240.2817 ± 5 ppm) from the full-scan high-resolution data. Although a chromatographic peak was observed in the retention time region expected for a C_17_ n-alkane, spectral deconvolution and library matching revealed that this signal corresponded to 2-isopropyl-5-[(E)-2-phenylethenyl]-1,3-benzenediol (C_17_H_18_O_2_; MW 254.13; CAS 79338-84-4), a phenolic compound with a molecular formula entirely distinct from that of heptadecane. The mass spectrum of this compound exhibited a base peak at *m*/*z* 239 and characteristic fragments at *m*/*z* 165, 115, and 202, which are inconsistent with the fragmentation pattern of linear alkanes. No signal attributable to the heptadecane molecular ion at the accurate mass level (*m*/*z* 240.2817) was detected in either sample. Third, the data acquired under low-energy electron ionization (LE-EI at 15 eV) were processed following the same approach. Under these soft ionization conditions, the molecular ion of n-alkanes is expected to be the dominant species in the mass spectrum, providing enhanced sensitivity for intact M^+^• detection. The EIC at *m*/*z* 240.2817 ± 5 ppm extracted from the 15 eV data confirmed the absence of any signal attributable to heptadecane in both samples. Hexane blanks showed no evidence of heptadecane contamination from the chromatographic system or solvents.

The absence of heptadecane in the volatile fraction of Brazilian green propolis is consistent with the comprehensive chemical characterization reported by Baptista Pereira et al. [17], who identified up to 60 compounds in green propolis essential oil by GC/FID/MS without detecting heptadecane among the annotated constituents. Although n-alkanes such as undecane have been reported as minor constituents of green propolis essential oils [17], heptadecane does not appear to be a characteristic component of the volatile fraction of Brazilian green propolis derived from *Baccharis dracunculifolia*. These findings indicate that the heptadecane detected as a differentially accumulated metabolite in the brain tissue of propolis-treated animals is unlikely to originate directly from the propolis employed in the study. Alternative sources or metabolic pathways should be considered in the interpretation of the metabolomic data.

## 3. Discussion

The present non-targeted metabolomic analysis demonstrates how standardized Brazilian green propolis alters striatal biochemistry in the 6-OHDA model of Parkinsonism. These integrated metabolic signatures not only reflect the neurotoxic impact of dopaminergic damage but also uncover adaptive and compensatory responses induced by propolis treatment. In summary, the results outline a mechanistic framework for interpreting the key metabolites discussed below and underscore their potential translational significance to PD.

From a biochemical perspective, the increase in gluconic acid in the striatum of dopaminergic-lesioned rats likely reflects a metabolic rerouting of glucose toward extramitochondrial oxidative pathways. Based on previous studies, this metabolic alteration may be associated with oxidative stress, mitochondrial dysfunction, and an increased NADPH requirement to maintain glutathione homeostasis [18,19]. D-Gluconic acid can be formed by enzymatic oxidation by glucose dehydrogenase or related oxidoreductases [20,21], but also by non-enzymatic oxidation under conditions enriched in reactive oxygen species (ROS) and labile iron, a biochemical environment characteristic of 6-OHDA-induced neurotoxicity [22]. The detected gluconic acid exists predominantly as gluconate, a physiologically relevant form with proven Fe^3+^ chelating capacity [23]. This chemical reactivity provides a plausible compensatory mechanism for buffering iron-induced oxidative damage in PD [24,25]. Therefore, the observed increase in gluconic acid probably represents both a marker of redox imbalance and a compensatory response to reduce iron-induced oxidative stress. The attenuation of this alteration by propolis treatment suggests that the compound modulates redox-sensitive metabolic nodes, in line with its reported effects on oxidative and metabolic homeostasis [12,26,27,28]. These results are consistent with emerging clinical and preclinical evidence linking shifts in glucose metabolism to oxidative stress in PD [29].

Among the metabolites contributing to group discrimination, heptadecane emerged as a particularly relevant feature due to its significant increase in propolis-treated animals compared with the untreated Parkinsonian group. Because long-chain hydrocarbons have previously been reported within the volatile fraction of propolis [30,31], the detection of heptadecane in striatal tissue initially raised the possibility that this metabolite could derive directly from the administered extract. To address this question, we performed a complementary high-resolution GC-Q-TOF characterization of both raw Brazilian green propolis and the standardized formulation used for oral gavage. Notably, heptadecane was not detected in either preparation, even after targeted screening under analytical conditions optimized for molecular ion preservation and exact-mass detection. These findings substantially refine the interpretation of the metabolomic dataset by demonstrating that the differential accumulation of heptadecane in the striatum cannot be directly attributed to the chemical composition of the administered propolis itself.

The integration of untargeted metabolomics with targeted chemical characterization represents an important strength of the present study, as it enabled a more rigorous interpretation of discriminant metabolites identified in brain tissue. Rather than reflecting passive incorporation of exogenous constituents, the observed metabolic alterations appear to represent broader biological responses associated with propolis supplementation in the 6-OHDA model. The consistent discrimination of heptadecane between experimental groups supports its relevance as a metabolomic feature associated with propolis exposure, even though its precise biochemical origin remains unresolved. Collectively, these findings highlight the complexity of metabolic remodeling in the Parkinsonian striatum and underscore the value of combining high-resolution chemical profiling with tissue metabolomics to strengthen biological interpretation in studies involving natural bioactive compounds.

Suberic acid, a medium-chain dicarboxylic acid, emerged as one of the most prominently altered metabolites in the present dataset. The accumulation of dicarboxylic acids is a well-established feature of conditions involving impaired mitochondrial fatty acid β-oxidation, in which compensatory activation of peroxisomal and microsomal ω-oxidation pathways contributes to their generation [32]. In the 6-OHDA model, striatal levels of suberic acid were markedly elevated, a metabolic signature consistent with disruptions in lipid catabolism and mitochondrial stress frequently reported in experimental and clinical contexts of PD. Importantly, treatment with Brazilian green propolis significantly attenuated the enrichment of suberic acid observed in the Parkinsonian group. Although the present study did not directly assess β-oxidation enzyme activity, peroxisomal function, or acylcarnitine profiles, the modulation of suberic acid levels indicates a shift in the lipid metabolic landscape associated with the 6-OHDA lesion. These findings do not support a direct inference of restored β-oxidation; rather, they point to a metabolic profile compatible with improved regulation of fatty acid turnover and mitochondrial–peroxisomal lipid handling. Beyond mechanistic considerations, suberic acid has been identified as a discriminant metabolite in clinical metabolomic studies of PD, underscoring its potential translational relevance as a marker of altered lipid metabolism [33]. In this context, the consistent modulation of suberic acid observed here reinforces the concept that lipid catabolism-related metabolic signatures may serve as sensitive indicators of neurodegenerative processes and their response to intervention.

A significant increase in tartaric acid was observed in the striatum of propolis-treated Parkinsonian rats. Tartaric acid is a plant-derived organic acid commonly found in dietary sources such as grapes and tamarind. Its detection in brain tissue following propolis treatment is consistent with the incorporation or metabolic processing of exogenous small organic acids within the central nervous system. Previous studies have shown that tartaric acid and related compounds can influence cellular pathways linked to redox balance and vascular function, including AMPK- and eNOS/NO/cGMP-associated signaling, as well as exhibiting antioxidant and vasorelaxant properties [34,35]. In addition, D-tartrate analogues have been reported to modulate dopaminergic vesicular transport [36], suggesting potential relevance to dopaminergic systems. Organic anion transporters (OATs), which mediate the exchange of small organic acids across biological membranes, including the blood–brain barrier, may contribute to the cerebral handling of compounds within this class [37]. However, the present study did not directly assess transporter activity, intracellular signaling pathways, or neurotransmitter dynamics. Therefore, the mechanistic basis underlying the increase in tartaric acid remains to be determined. Within these constraints, the consistent elevation of this metabolite in propolis-treated animals supports its relevance as a discriminant feature of the metabolic profile associated with the intervention, warranting further investigation into its origin and potential functional implications.

Although the primary focus of the present study was the characterization of the striatal metabolomic landscape, the integration of behavioral and histological data provides a coherent and biologically consistent framework supporting the effects of Brazilian green propolis in experimental Parkinsonism. Importantly, the observed metabolic remodeling occurred in parallel with robust functional and histological outcomes. Propolis-treated animals exhibited significant improvement in motor performance in the rotarod test, indicating attenuation of 6-OHDA-induced motor deficits. This behavioral effect was accompanied by attenuation of nigrostriatal dopaminergic neurodegeneration, as demonstrated by tyrosine hydroxylase immunohistochemistry in both the substantia nigra pars compacta and the striatum. The convergence of these findings supports the interpretation that the observed functional improvement is associated with attenuation of dopaminergic neurodegeneration within the nigrostriatal pathway.

**Figure 7:**
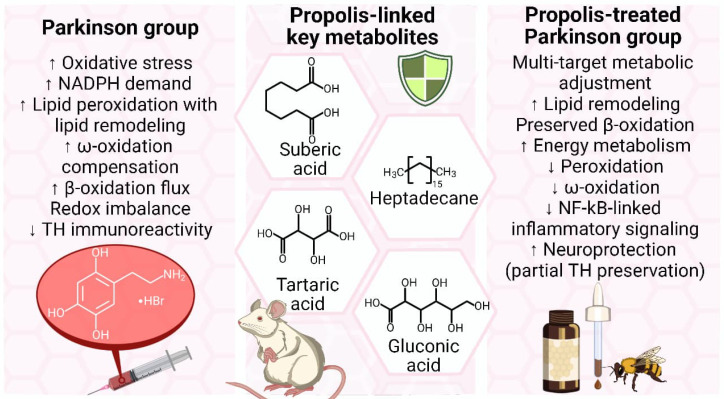
Overview of striatal metabolic alterations induced by 6-OHDA lesion and their modulation by Brazilian green propolis. The 6-OHDA model is characterized by oxidative stress, increased NADPH demand, dopaminergic fiber loss, and impaired mitochondrial β-oxidation with compensatory peroxisomal ω-oxidation, leading to the accumulation of suberic and gluconic acids. Propolis treatment mitigates these alterations, being associated with preserved β-oxidation, reduced lipid peroxidation, attenuation of NF-κB-driven inflammatory signaling, and remodeling of lipid and energy metabolism. Metabolite changes, including altered tartaric acid and heptadecane levels and reduced suberic acid, reflect coordinated metabolic alterations associated with propolis treatment, consistent with multi-target neuroprotective effects in PD. Upward arrows (↑) indicate increased biological processes, whereas downward arrows (↓) indicate decreased biological processes.

The neuroprotective profile observed may be partially related to the complex polyphenolic composition of Brazilian green propolis, which contains multiple bioactive constituents with reported antioxidant and neurobiological Properties (Figure 7). Compounds such as chrysin and galangin have been previously shown to attenuate dopaminergic neurodegeneration and improve motor outcomes in the 6-OHDA model, effects associated with modulation of oxidative stress and endogenous antioxidant pathways, including Nrf2/Keap1 signaling [38,39,40]. Nevertheless, as the present study employed a standardized whole extract rather than isolated compounds, these mechanisms should be interpreted as biologically plausible contributors rather than definitive mediators of the effects observed. Collectively, these findings indicate that Brazilian green propolis is associated with an integrated metabolic and neurobiological response in the 6-OHDA model, accompanied by attenuation of dopaminergic dysfunction and improved motor performance.

### Translational, Safety, Dose-Relevance and Clinical Significance

The standardized Brazilian green propolis extract (EPP-AF^®^-C; Apis Flora, Ribeirão Preto, Brazil) employed in this study has been extensively characterized for chemical reproducibility, safety, and biological consistency under Good Manufacturing Practice (GMP) standards. Its defined phenolic and flavonoid profile underpins well-documented antioxidant, anti-inflammatory, and immunomodulatory activities [41].

The oral dosing regimen (200 mg/kg/day for 28 days) was selected based on prior studies from our group, which demonstrated robust neuroprotective efficacy of this same standardized extract in the 6-OHDA model of Parkinsonism [15,16], including preservation of nigrostriatal dopaminergic neurons and attenuation of oxidative stress. According to FDA-recommended body surface area conversion, this corresponds to a human-equivalent dose of approximately 32 mg/kg/day.

Clinical trials employing standardized Brazilian green propolis extracts have consistently reported excellent tolerability, even under chronic oral intake up to 800–900 mg/day, with no hematological, hepatic, or renal toxicity [42,43,44]. In line with these data, no signs of behavioral distress, weight loss, gastrointestinal alteration, or systemic toxicity were observed in our study, reinforcing the safety of prolonged oral administration.

The convergence of preclinical and clinical safety data strengthens the translational feasibility of this intervention and underscores the pharmacological efficacy of propolis-based adjuvant strategies in neurodegenerative diseases. Furthermore, the use of a chemically standardized extract ensures batch-to-batch reproducibility, thus eliminating a critical bottleneck in the translational development of natural products.

In summary, these results establish a solid continuum from experimental to clinical and support the safe, rational, and mechanistically sound application of Brazilian green propolis as a neuroprotective nutraceutical.

## 4. Materials and Methods

### 4.1. Animals and Ethical Approval

Male Wistar rats (8 weeks old, 230–300 g) were used. All procedures adhered to the Brazilian Society for Laboratory Animal Science (SBCAL) guidelines and were approved by the Ethics Committee on Animal Use of the Federal University of São Paulo (CEUA no. 9410140323). The reuse of cryopreserved biological material was authorized under protocol no. 2164030225. Rats were housed in groups of four under controlled conditions (12 h light/dark cycle, 22 ± 2 °C, 55 ± 5% humidity) with ad libitum access to food and water.

### 4.2. Experimental Design and Groups

**Figure 8:**
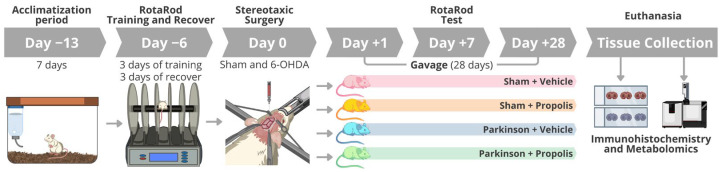
Experimental timeline and study design. Rats underwent a 7-day acclimatization period (Day −13), followed by rotarod training and recovery (Day −6). On Day 0, animals were subjected to stereotaxic surgery with bilateral intrastriatal injections of 6-hydroxydopamine (6-OHDA) or vehicle (Sham). Starting on Day +1, animals received daily oral gavage with standardized Brazilian green propolis (200 mg/kg) or vehicle for 28 consecutive days. Motor performance was assessed using the rotarod test on Day +7. Four experimental groups were evaluated: Sham + Vehicle, Sham + Propolis, Parkinson + Vehicle, and Parkinson + Propolis. At Day +28, animals were euthanized, and brain tissues were collected for immunohistochemistry and untargeted GC–MS-based metabolomic analyses.

Following a 7-day acclimatization, rats underwent stereotaxic surgery (day 0) and received daily treatments for 28 days (days 1–28) (Figure 8). A total of 64 animals were used: 32 for immunohistochemistry (*n* = 8 per group) and 32 for metabolomics (*n* = 8 per group). Animals were randomly assigned to four experimental groups. Group 1: Sham + Vehicle (water): bilateral striatal vehicle injections (0.9% saline with 0.3% ascorbic acid) and oral gavage with filtered water. Group 2: Sham + Propolis: vehicle injections followed by oral gavage with standardized green propolis extract (EPP-AF^®^-C). Group 3: Parkinson + Vehicle: bilateral 6-hydroxydopamine (6-OHDA) injections followed by oral gavage with filtered water. Group 4: Parkinson + Propolis: 6-OHDA injections followed by oral gavage with EPP-AF^®^-C. For metabolomics analysis, samples identified as outliers during quality control and statistical evaluation were excluded, yielding a final *n* = 6 per group.

An additional cohort of animals was used for behavioral assessment using the rotarod test. This experiment was conducted with 41 animals and was approved by the Ethics Committee on Animal Use of the Federal University of São Paulo (CEUA no. 7848020920). Animals were distributed into the following groups: Sham + Vehicle (*n* = 11), Sham + Propolis (*n* = 10), Parkinson + Vehicle (n = 10), and Parkinson + Propolis (*n* = 10). The rotarod test was used to evaluate motor coordination and balance as functional outcomes of the treatments. Animals were housed under controlled environmental conditions (22 ± 2 °C, 12 h light/dark cycle, lights on at 7:00 a.m.) with ad libitum access to food and water. Upon arrival, animals were allowed to acclimatize to the animal facility for at least 5–7 days prior to the initiation of experimental procedures. To minimize variability associated with motor learning and to establish a stable baseline, animals underwent rotarod pre-training over three consecutive days (one session per day). A 72 h interval was allowed between the final training session and stereotaxic surgery for 6-OHDA administration to avoid potential confounding effects of stress or fatigue and to ensure recovery before lesion induction. Following lesion induction, animals received daily treatment with standardized Brazilian green propolis or vehicle (water) for six consecutive days. Behavioral assessment was conducted 7 days after lesion induction to evaluate motor impairment and the effects of the intervention.

### 4.3. Stereotaxic 6-OHDA Lesion of the Striatum

To induce dopaminergic neurodegeneration, rats were anesthetized with ketamine (100 mg/kg, i.p.) and xylazine (10 mg/kg, i.p.) and secured in a stereotaxic frame (Insight EFF-331). Following scalp incision and skull exposure, 6-hydroxydopamine hydrochloride (6-OHDA; 12 µg/µL in 0.3% ascorbic acid; Sigma-Aldrich, St. Louis, MO, USA) was injected bilaterally into the striatum using a 10 µL Hamilton syringe. Each animal received four injections (1 µL each), two per hemisphere, at the following stereotaxic coordinates [45]: (1) Lateral: −2.7 mm, AP: Bregma, DV: −4.5 mm; (2) Lateral: −3.2 mm, AP: +0.5 mm, DV: −4.5 mm; (3) Lateral: +2.7 mm, AP: Bregma, DV: −4.5 mm; and (4) Lateral: +3.2 mm, AP: +0.5 mm, DV: −4.5 mm. Injections were delivered at 0.2 µL/min, with an 8 min dwell time before needle withdrawal to prevent reflux. Sham animals received vehicle injections under identical conditions. Postoperatively, rats were monitored daily for hydration, grooming, and locomotor activity.

### 4.4. Oral Administration of Standardized Brazilian Green Propolis

**Figure 9:**
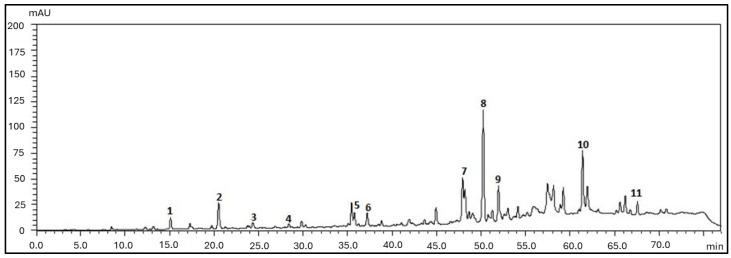
Representative HPLC chromatogram of standardized Brazilian green propolis extract (EPP-AF^®^-C, Apis Flora, Ribeirão Preto, Brazil). The chromatogram shows the characteristic fingerprint of EPP-AF^®^-C, highlighting the major phenolic and flavonoid markers identified and quantified: (**1**) caffeic acid, (**2**) p-coumaric acid, (**3**) 3,5-dicaffeoylquinic acid, (**4**) 4,5-dicaffeoylquinic acid, (**5**) cinnamic acid, (**6**) aromadendrin, (**7**) drupanin, (**8**) chrysin, (**9**) galangin, (**10**) artepillin C, and (**11**) baccharin. The chemical profile corresponds to the standardized composition previously reported for this extract [40].

The standardized Brazilian green propolis extract (EPP-AF^®^-C, 80 mg/mL; Apis Flora Co., Ribeirão Preto, Brazil), primarily derived from *Baccharis dracunculifolia* (Asteraceae), was used in this study. Chemical standardization ensures batch-to-batch reproducibility, addressing a major limitation in the translational development of natural products. To further guarantee experimental consistency, a single production lot of EPP-AF^®^-C was employed throughout the study. The extract was administered by oral gavage at a dose of 200 mg/kg/day for 28 consecutive days, beginning 24 h after surgery. The extract was diluted in filtered water, and the administration volume was adjusted weekly according to the animals’ body weight to ensure accurate dosing. The selected dose was based on previous studies [15,16] demonstrating safety and neuroprotective efficacy in rodent models of neurodegeneration. The extract contains 38.27% total phenolics and 8.69% total flavonoids, expressed in galangin and rutin equivalents, respectively. Quantitative HPLC-DAD analysis demonstrated that each gram of EPP-AF^®^-C powder contained 2.04 mg of caffeic acid, 3.21 mg of 3,5-dicaffeoylquinic acid, 1.71 mg of 4,5-dicaffeoylquinic acid, 3.88 mg of aromadendrin-O′-methyl ether, 10.04 mg of chrysin, 7.30 mg of galangin, 9.12 mg of drupanin, 24.30 mg of artepillin C, and 2.65 mg of baccharin [41]. Chromatographic profiles and compound quantification were confirmed using authentic external standards. The representative chromatogram illustrates (Figure 9) the characteristic fingerprint of the standardized extract, as defined by Apis Flora quality specifications and previous reports. Sham groups received filtered water by gavage under the same schedule.

### 4.5. Tissue Collection for Immunohistochemistry and Metabolomics

For immunohistochemistry, animals were deeply anesthetized and transcardially perfused with 50 mL of PBS (0.1 M, pH 7.4) followed by 200–300 mL of 4% paraformaldehyde (pH 7.4). Brains were collected, post-fixed overnight at 4 °C, and cryoprotected in 30% sucrose. For metabolomics, animals were euthanized under deep anesthesia without perfusion. Striata were rapidly dissected from fresh brains, snap-frozen on dry ice, and stored at −80 °C until analysis.

### 4.6. Rotarod Test

Motor coordination and balance were assessed using the rotarod test (Insight^®^ EFF-412, Ribeirão Preto, Brazil), a widely used assay for evaluating nigrostriatal-dependent motor performance in experimental Parkinsonism. Prior to stereotaxic surgery for 6-OHDA administration, animals underwent a pre-training phase to minimize variability associated with motor learning and to ensure stable baseline performance. Training was conducted over three consecutive days (one session per day), following a 60 min acclimatization period to the experimental room to reduce stress-related confounding effects. During training, the rotarod was operated at a constant speed of 12 rpm, and animals were required to remain on the rotating rod for 60 s. Each session consisted of up to three trials. Animals that failed to maintain balance for at least 60 s in the final training session were excluded from the study. Motor performance was evaluated 7 days after 6-OHDA-induced dopaminergic lesion, following 6 consecutive days of treatment with standardized Brazilian green propolis or vehicle (water), allowing assessment of the functional impact of the intervention. Testing was performed using an accelerating rotarod protocol. Animals were placed on the rotating rod, and rotation speed was progressively increased across five successive levels (16, 20, 25, 28, and 37 rpm) over a 5 min period (maximum test duration). The latency to first fall and the total number of falls were recorded. All behavioral assessments were conducted during the light phase of the light–dark cycle in a quiet room under controlled environmental conditions. To prevent olfactory cue interference, the apparatus was cleaned with 20% ethanol between sessions.

#### Statistical Analysis

To evaluate the main effects of lesion and treatment, as well as their interaction, a generalized linear model (GLM) with gamma distribution and logarithmic link function was applied. This approach is appropriate for continuous, strictly positive data with heterogeneous variance, as typically observed for rotarod latency measurements. The significance of the interaction term was assessed using a likelihood-ratio F-test. All analyses were performed in the R statistical environment (R version 4.3.1, 16 June 2023). Given the presence of a significant interaction, planned pairwise comparisons were conducted using the Mann–Whitney U test (Wilcoxon rank-sum test), with Bonferroni correction applied across four comparisons: Sham + Vehicle vs. Parkinson + Vehicle; Parkinson + Vehicle vs. Parkinson + Propolis; Sham + Vehicle vs. Parkinson + Propolis; and Sham + Vehicle vs. Sham + Propolis. Effect size was estimated using Rosenthal’s r (r = |z|/√N) and interpreted as small (r ≥ 0.1), medium (r ≥ 0.3), or large (r ≥ 0.5). The significance level was set at α = 0.05 for all analyses. Graphical representations of the data were generated using GraphPad Prism version 10.0 (GraphPad Software, San Diego, CA, USA).

### 4.7. Tyrosine Hydroxylase Immunohistochemistry

Tyrosine hydroxylase immunohistochemistry was performed to validate the 6-OHDA model and to demonstrate the neuroprotective effects of propolis treatment. To evaluate dopaminergic neuron loss in the substantia nigra pars compacta (SNpc) as well as striatal fiber degeneration, perfused brains were cryoprotected in 30% sucrose and coronally sectioned at 40 μm using a Microm HM 505E cryostat (Microm International GmbH, Walldorf, Germany). Free-floating sections were incubated overnight at 4 °C with anti-TH primary antibody (1:1000, Millipore, Burlington, MA, USA), followed by HRP-conjugated secondary antibody (anti-rabbit, 1:2000, Calbiochem®, San Diego, CA, USA). Staining was visualized using 3,3′-diaminobenzidine (DAB) in Tris-HCl buffer. Sections were mounted, dehydrated, cleared in xylene, and coverslipped. Images were acquired using a Nikon Eclipse E600 microscope (Nikon Corporation, Tokyo, Japan) and analyzed using ImageJ software version 1.54 (National Institutes of Health, Bethesda, MD, USA). Quantification was performed using a field-based approach rather than stereological counting. For SNpc neuronal analysis, TH-positive neurons were counted in coronal sections sampled at 200 μm intervals across the rostrocaudal extent of the SNpc. For striatal analysis, optical density of TH-immunoreactive fibers was measured in standardized regions of interest within the striatum. Measurements from multiple sections were averaged for each animal prior to statistical analysis.

#### Statistical Analysis

Quantitative data from tyrosine hydroxylase (TH) immunohistochemistry, including striatal fiber density and neuronal counts in the substantia nigra pars compacta (SNpc), were expressed as mean ± standard error of the mean (SEM). Statistical comparisons among experimental groups were performed using two-way analysis of variance (ANOVA) to evaluate the main effects of lesion and treatment, followed by Tukey’s post hoc test to identify specific between-group differences. Statistical significance was set at *p* < 0.05. All analyses were conducted using GraphPad Prism (v.10.0; GraphPad Software, USA).

### 4.8. Untargeted GC–MS Metabolomic Profiling of Striatal Tissue

Striatal tissues were rapidly dissected from fresh brains, snap-frozen on dry ice, and stored at −80 °C until analysis. For metabolomic profiling, 10–15 mg of tissue were homogenized in methanol/water (1:4, *v*/*v*) using metal beads in a TissueLyser system (Qiagen, Hilden, Germany) and centrifuged; supernatants were evaporated to dryness in a SpeedVac concentrator (Thermo Fisher Scientific, Waltham, MA, USA). Metabolites were derivatized sequentially via methoximation (O-methoxyamine hydrochloride in pyridine) followed by silylation (BSTFA + 1% TMCS), with heptane containing pentadecanoic acid as internal standard. Pooled quality control (QC) samples, prepared by combining aliquots of all extracts, and reagent blanks were interspersed throughout the analytical sequence to monitor instrumental stability, analytical reproducibility, and signal drift. Sample injections were randomized to minimize batch effects and ensure analytical robustness [46]. GC–MS analyses were performed on a Shimadzu GCMS-QP2020NX system (Shimadzu Corporation, Kyoto, Japan) equipped with an SLB-5ms fused silica capillary column (30 m × 0.25 mm, 0.25 μm; Sigma-Aldrich, St. Louis, MO, USA)) and an AOC-20i autosampler (Shimadzu Corporation, Kyoto, Japan). Helium was used as carrier gas with a column flow of 1.10 mL/min and a total flow of 17.0 mL/min. The oven temperature program ranged from 40 °C to 320 °C at 10 °C/min with a final hold of 8 min. Injector and interface temperatures were maintained at 250 °C, and the ion source temperature was 230 °C. Electron impact ionization was applied at 70 eV with full scan acquisition in the *m*/*z* range of 40–650. Raw chromatographic data were processed using LabSolutions software (v.4.5) for baseline correction, peak deconvolution, retention time alignment, and integration. Features detected in reagent blanks were excluded, and only metabolites consistently detected across samples and QC injections were retained. Peak areas were normalized to the internal standard to correct for analytical variability [47,48]. Metabolite annotation was performed by comparison of mass spectra and retention indices against the NIST17 library, GCMS Solution spectral library, and the Smart Metabolite Database. Identification followed the Metabolomics Standards Initiative (MSI) guidelines, including Level 1 identification when confirmed with authentic standards and Level 2 annotation based on spectral similarity and retention index matching [44,45]. Analytical reproducibility was evaluated using QC injections distributed throughout the analytical run. All metabolites considered for statistical analysis exhibited coefficients of variation below 30% in QC samples, and the overall relative standard deviation (RSD) of QC injections across the experiment was 8.77%, confirming the stability and robustness of the GC–MS analytical platform.

#### Statistical Analysis

Processed metabolomic data were imported into MetaboAnalyst 6.0 for statistical analysis. Data were median-normalized, log-transformed, and Pareto-scaled prior to multivariate analysis. Principal component analysis (PCA) was initially applied as an unsupervised exploratory approach to assess the global data structure, sample distribution, and clustering of quality control (QC) samples. This analysis allowed evaluation of analytical reproducibility and identification of potential outliers within the dataset. Samples identified as outliers during multivariate inspection and complementary machine-learning evaluation were excluded prior to final modeling. Following exclusion of these samples, the dataset was re-normalized and re-scaled before subsequent uni- and multivariate analyses. Supervised multivariate modeling was then performed using partial least squares discriminant analysis (PLS-DA) to enhance group discrimination and identify metabolites contributing to experimental group separation. Variable importance in projection (VIP) scores were calculated to rank metabolites according to their contribution to the model. Model performance and predictive capacity were evaluated using cross-validation parameters, including classification accuracy, coefficient of determination (R^2^), and predictive ability (Q^2^). Model robustness was further assessed by permutation testing using 100 permutations to evaluate the risk of model overfitting. Univariate statistical analysis was conducted using one-way analysis of variance (ANOVA) followed by Tukey’s post hoc test to identify pairwise group differences. Multiple comparisons were corrected using the Benjamini–Hochberg false discovery rate (FDR) procedure. Metabolites with significant adjusted *p*-values were considered discriminant. Metabolomic results are presented as PCA and PLS-DA score plots, VIP analysis, model validation metrics, and boxplots illustrating the relative abundance of significantly altered metabolites among experimental groups.

### 4.9. GC-Q-TOF: Targeted Investigation of Heptadecane

#### 4.9.1. Chemicals and Materials

Hexane (GC grade) was used as an extraction solvent and for sample dilution. Distilled water was employed for hydrodistillation. Anhydrous sodium sulfate (Na_2_SO_4_) was used as a drying agent. Nylon membrane filters (0.45 µm) were used for sample filtration prior to injection.

#### 4.9.2. Sample Preparation

##### Raw Propolis (Hydrodistillation)

Raw green propolis (Lot 067400626) was ground and sieved through a 12-mesh sieve (1.70 mm). A mass of 1000.29 g of the ground propolis was transferred to a 1000 mL round-bottom flask, and 500 mL of distilled water was added. The sample was subjected to steam distillation using a Clevenger-type apparatus for 5 h after the onset of boiling. At the end of the distillation, 0.2 mL of essential oil was collected. Subsequently, 1.0 mL of GC-grade hexane was added to the Clevenger arm to solubilize and recover the volatile compounds. The mixture was kept in a freezer to allow the aqueous phase to freeze and separate. After phase separation, the supernatant (1.0 mL) was collected and filtered through a 0.45 µm membrane. Prior to analysis, the sample was diluted 1:10 (*v*/*v*) in hexane (100 µL of sample + 900 µL of hexane).

##### Propolis Formulation (Liquid–Liquid Extraction)

The gavage formulation containing 10% (*w*/*v*) of propolis soft extract (80% dry mass) and 10% (*w*/*v*) of castor oil was subjected to liquid–liquid extraction (LLE). A volume of 20 mL of the formulation was transferred to a separating funnel, and 10 mL of GC-grade hexane was added. The mixture was vigorously shaken for 5 min with periodic pressure relief. After phase separation, the organic phase (upper hexane layer) was collected. The extraction procedure was repeated twice with 10 mL of fresh hexane each time. The combined organic fractions were centrifuged at 3000 rpm for 10 min to ensure complete phase separation. The extract was concentrated to dryness using a speed vacuum concentrator and subsequently reconstituted in 1.0 mL of hexane and filtered through a 0.45 µm membrane. Prior to analysis, the sample was diluted 1:50 (*v*/*v*) in hexane (20 µL of sample + 980 µL of hexane).

#### 4.9.3. Instrumental Analysis

The volatile fraction of both propolis samples was analyzed using an Agilent 7890B gas chromatograph coupled to an Agilent 7250 Accurate-Mass Quadrupole Time-of-Flight mass spectrometer (GC-Q-TOF) equipped with an electron ionization (EI) source (Agilent Technologies, Santa Clara, CA, USA). Chromatographic separation was performed on an HP-5ms Ultra Inert capillary column (15 m × 0.25 mm i.d. × 0.25 µm film thickness; 5% phenyl methylpolysiloxane; Agilent Technologies). Helium (purity ≥ 99.999%) was used as carrier gas at a constant flow rate of 1.0 mL/min. The injection volume was 1.0 µL in split mode (1:20 for raw propolis; 1:50 for the gavage formulation). The inlet temperature was set at 250 °C, and the GC–MS transfer line was maintained at 280 °C. The oven temperature program was as follows: initial temperature of 50 °C held for 2 min, ramped at 5 °C/min to 180 °C, then ramped at 10 °C/min to 280 °C and held for 3 min, resulting in a total run time of 41 min. A solvent delay of 3.5 min was applied. Two acquisition methods were employed for each sample: Method 1—Standard EI (70 eV). The mass spectrometer was operated in full-scan mode with standard electron ionization at 70 eV. The ion source temperature was set at 230 °C. Data were acquired over a mass range of *m*/*z* 40–500 at an acquisition rate of 5 spectra/s. This method was used for untargeted profiling of the volatile fraction and targeted screening for heptadecane by extracted ion chromatogram (EIC) at *m*/*z* 240.2817 ± 5 ppm. Method 2—Low-Energy EI (15 eV). The mass spectrometer was operated in full-scan mode with low-energy electron ionization (LE-EI) at 15 eV. The ion source temperature was set at 230 °C. Data were acquired over a mass range of *m*/*z* 40–500 at an acquisition rate of 5 spectra/s. The chromatographic conditions were identical to Method 1. The reduced ionization energy minimizes molecular fragmentation, thereby enhancing the relative abundance of the molecular ion (M^+^•), which facilitates unambiguous confirmation of compound identity through accurate mass measurement. Hexane blanks were injected before and after each sample under the same conditions to monitor potential carryover and system contamination.

## 5. Conclusions

In summary, untargeted GC–MS identified a defined set of discriminant metabolites (gluconic acid, heptadecane, suberic acid, and tartaric acid) that characterize the striatal metabolic profile associated with Brazilian green propolis in the 6-OHDA model, with links to redox balance, energy metabolism, and mitochondrial–peroxisomal lipid oxidation. Among these, suberic acid emerged as a particularly relevant feature, showing consistent modulation between experimental groups and reinforcing its potential translational significance. The modulation of suberic acid levels, together with the established safety profile of the standardized extract (EPP-AF^®^), further supports the translational relevance of these findings.

These metabolic alterations were accompanied by attenuation of nigrostriatal dopaminergic neurodegeneration and improved motor performance in the rotarod test, indicating that Brazilian green propolis engages integrated metabolic, histological, and functional processes in experimental Parkinsonism.

## Figures and Tables

**Table 1:** PLS cross-validation performance after outlier removal. The PLS model, including outliers, is shown in the Supplementary Materials.

Metric	Before Outlier Removal	After Outlier Removal
Accuracy	0.23	0.63
R^2^	0.67	0.90
Q^2^	0.35	0.65

**Table 2:** Results of one-way ANOVA and post hoc analysis identifying metabolites significantly different among experimental groups. The table shows the F statistic (F value), raw *p*-values, −log10(*p*-value) used for visualization of statistical significance, and false discovery rate (FDR)-adjusted *p*-values. Significant pairwise differences between groups were further evaluated using Tukey’s honestly significant difference (HSD) post hoc test, with the corresponding group comparisons indicated for each metabolite.

Metabolite	F Value	*p* Value	−log10(*p*)	FDR	Tukey’s HSD Comparisons
Suberic acid	10.862	0.00018947	3.7225	0.0083365	PKV–PKP, SHP–PKP, SHV–PKP
Heptadecane	7.1186	0.0019369	2.7129	0.042613	PKV–PKP, SHP–PKV
Tartaric acid	6.0227	0.0042811	2.3684	0.0498	PKV–PKP, SHP–PKP, SHV–PKP
Gluconic acid	5.9485	0.0045272	2.3442	0.0498	SHV–PKP, SHV–PKV

## Data Availability

The datasets generated and/or analyzed during the current study are available from the corresponding author upon reasonable request.

## Supplementary Materials

The following supporting information can be downloaded at: https://www.mdpi.com/article/10.3390/molecules31111791/s1. Figure S1: Multivariate and univariate metabolomic analyses before and after outlier removal. Table S1: Metabolites detected in untargeted GC–MS metabolomic analysis of striatal tissue across the experimental groups. Table S2: Quality control (QC) injections used to evaluate the analytical stability and reproducibility of the GC–MS platform during the metabolomic analysis.

## Abbreviations

6-OHDA	6-Hydroxydopamine
AMPK	AMP-Activated Protein Kinase
ANOVA	Analysis of Variance
BBB	Blood–Brain Barrier
BSTFA	N,O-Bis(trimethylsilyl)trifluoroacetamide
CEUA	Ethics Committee on Animal Use
cGMP	Cyclic Guanosine Monophosphate
DAB	3,3′-Diaminobenzidine
EPP-AF^®^-C	Standardized Brazilian Green Propolis Extract
FDR	False Discovery Rate
GC–MS	Gas Chromatography-Mass Spectrometry
HMDB	Human Metabolome Database
HPLC-DAD	High-Performance Liquid Chromatography with Diode-Array Detection
HRP	Horseradish Peroxidase
NADPH	Nicotinamide Adenine Dinucleotide Phosphate
NF-kB	Nuclear Factor kappa B
NO	Nitric Oxide
OATs	Organic Anion Transporters
PBS	Phosphate-Buffered Saline
PCA	Principal Component Analysis
PD	Parkinson’s Disease
PLS-DA	Partial Least Squares-Discriminant Analysis
QC	Quality Control
ROS	Reactive Oxygen Species
SEM	Standard Error of the Mean
SHV	Sham + Vehicle
SHP	Sham + Propolis
SNpc	Substantia Nigra pars compacta
TH	Tyrosine Hydroxylase
TMCS	Trimethylchlorosilane
VIP	Variable Importance in Projection

## References

[1] Chung S.J., Yoo H.S., Oh J.S., Kim J.S., Ye B.S., Sohn Y.H., Lee P.H. (2018). Effect of Striatal Dopamine Depletion on Cognition in de Novo Parkinson’s Disease. Park. Relat. Disord..

[2] Jeong S.H., Park C.W., Lee H.S., Kim Y.J., Yun M., Lee P.H., Sohn Y.H., Chung S.J. (2023). Patterns of Striatal Dopamine Depletion and Motor Deficits in de Novo Parkinson’s Disease. J. Neural Transm..

[3] Li X., Bu S., Pang H., Yu H., Zhao M., Wang J., Liu Y., Fan G. (2025). Mapping Striatal Functional Gradients and Associated Gene Expression in Parkinson’s Disease with Continuous Cognitive Impairment. npj Park. Dis..

[4] Weintraub D., Aarsland D., Biundo R., Dobkin R., Goldman J., Lewis S. (2022). Management of Psychiatric and Cognitive Complications in Parkinson’s Disease. BMJ.

[5] Lu Y., Zhang X., Zhao L., Yang C., Pan L., Li C., Liu K., Bai G., Gao H., Yan Z. (2018). Metabolic Disturbances in the Striatum and Substantia Nigra in the Onset and Progression of MPTP-Induced Parkinsonism Model. Front. Neurosci..

[6] Yang C., Zhang T., Wang W., Xiang Y., Huang Q., Xie C., Zhao L., Zheng H., Yang Y., Gao H. (2020). Brain-Region Specific Metabolic Abnormalities in Parkinson’s Disease and Levodopa-Induced Dyskinesia. Front. Aging Neurosci..

[7] Kalia L.V., Lang A.E. (2015). Parkinson’s Disease. Lancet.

[8] Poewe W., Seppi K., Tanner C.M., Halliday G.M., Brundin P., Volkmann J., Schrag A.-E., Lang A.E. (2017). Parkinson Disease. Nat. Rev. Dis. Primers.

[9] Ungerstedt U. (1968). 6-Hydroxy-Dopamine Induced Degeneration of Central Monoamine Neurons. Eur. J. Pharmacol..

[10] Simola N., Morelli M., Carta A.R. (2007). The 6-Hydroxydopamine Model of Parkinson’s Disease. Neurotox. Res..

[11] Bové J., Perier C. (2012). Neurotoxin-Based Models of Parkinson’s Disease. Neuroscience.

[12] Takashima M., Ichihara K., Hirata Y. (2019). Neuroprotective Effects of Brazilian Green Propolis on Oxytosis/Ferroptosis in Mouse Hippocampal HT22 Cells. Food Chem. Toxicol..

[13] Xu X., Yang B., Wang D., Zhu Y., Miao X., Yang W. (2020). The Chemical Composition of Brazilian Green Propolis and Its Protective Effects on Mouse Aortic Endothelial Cells against Inflammatory Injury. Molecules.

[14] Vieira A.L.S., Correia V.T.V., Ramos A.L.C.C., Silva N.H.A., Jaymes L.A.C., Melo J.O.F., Paula A.C.C.F.F., Garcia M.A.V.T., Araújo R.L.B. (2023). Evaluation of the Chemical Profile and Antioxidant Capacity of Green, Brown, and Dark Propolis. Plants.

[15] Gonçalves V.C., Pinheiro D.J.L.L., de la Rosa T., de Almeida A.-C.G., Scorza F.A., Scorza C.A. (2020). Propolis as a Potential Disease-Modifying Strategy in Parkinson’s Disease: Cardioprotective and Neuroprotective Effects in the 6-OHDA Rat Model. Nutrients.

[16] Gonçalves V.C., Silva da Fonseca V., de Paula Faria D., Izidoro M.A., Berretta A.A., de Almeida A.-C.G., Fonseca F.L.A., Scorza F.A., Scorza C.A. (2022). Propolis Induces Cardiac Metabolism Changes in 6-Hydroxydopamine Animal Model: A Dietary Intervention as a Potential Cardioprotective Approach in Parkinson’s Disease. Front. Pharmacol..

[17] Pereira D.B., Alves N.S., Silva E.O., Epifanio N.M.M., Chaves D.S.A. (2024). Extraction Kinetics of Brazilian Green Propolis and Chemical Characterization of Its Volatiles. Chem. Biodivers..

[18] Rasmusson A.G., Soole K.L., Elthon T.E. (2004). Alternative NAD(P)H Dehydrogenases of Plant Mitochondria. Annu. Rev. Plant Biol..

[19] Stincone A., Prigione A., Cramer T., Wamelink M.M.C., Campbell K., Cheung E., Olin-Sandoval V., Grüning N., Krüger A., Alam M.T. (2015). The Return of Metabolism: Biochemistry and Physiology of the Pentose Phosphate Pathway. Biol. Rev..

[20] Umezawa K., Takeda K., Ishida T., Sunagawa N., Makabe A., Isobe K., Koba K., Ohno H., Samejima M., Nakamura N. (2015). A Novel Pyrroloquinoline Quinone-Dependent 2-Keto-D-Glucose Dehydrogenase from *Pseudomonas aureofaciens*. J. Bacteriol..

[21] Dai L., Jiang W., Jia R., Zhou X., Xu Y. (2022). Directional Enhancement of 2-Keto-Gluconic Acid Production from Enzymatic Hydrolysate by Acetic Acid-Mediated Bio-Oxidation with *Gluconobacter oxydans*. Bioresour. Technol..

[22] Valko M., Morris H., Cronin M. (2005). Metals, Toxicity and Oxidative Stress. Curr. Med. Chem..

[23] Ma Y., Li B., Zhang X., Wang C., Chen W. (2022). Production of Gluconic Acid and Its Derivatives by Microbial Fermentation: Process Improvement Based on Integrated Routes. Front. Bioeng. Biotechnol..

[24] Cheng R., Dhorajia V.V., Kim J., Kim Y. (2022). Mitochondrial Iron Metabolism and Neurodegenerative Diseases. Neurotoxicology.

[25] Levi S., Ripamonti M., Moro A.S., Cozzi A. (2024). Iron Imbalance in Neurodegeneration. Mol. Psychiatry.

[26] Saito Y., Tsuruma K., Ichihara K., Shimazawa M., Hara H. (2015). Brazilian Green Propolis Water Extract Up-Regulates the Early Expression Level of HO-1 and Accelerates Nrf2 after UVA Irradiation. BMC Complement. Altern. Med..

[27] Ni J., Wu Z., Meng J., Zhu A., Zhong X., Wu S., Nakanishi H. (2017). The Neuroprotective Effects of Brazilian Green Propolis on Neurodegenerative Damage in Human Neuronal SH-SY5Y Cells. Oxid. Med. Cell. Longev..

[28] Hirata Y., Motoyama M., Kimura S., Takashima M., Ikawa T., Oh-hashi K., Kamatari Y.O. (2021). Artepillin C, a Major Component of Brazilian Green Propolis, Inhibits Endoplasmic Reticulum Stress and Protein Aggregation. Eur. J. Pharmacol..

[29] Zhu A., Wu Z., Zhong X., Ni J., Li Y., Meng J., Du C., Zhao X., Nakanishi H., Wu S. (2018). Brazilian Green Propolis Prevents Cognitive Decline into Mild Cognitive Impairment in Elderly People Living at High Altitude. J. Alzheimers Dis..

[30] de Oliveira M.S., Cruz J.N., Ferreira O.O., Pereira D.S., Pereira N.S., Oliveira M.E.C., Venturieri G.C., Guilhon G.M.S.P., Souza Filho A.P.S., Andrade E.H.A. (2021). Chemical Composition of Volatile Compounds in *Apis mellifera* Propolis from the Northeast Region of Pará State, Brazil. Molecules.

[31] Isidorov V.A., Dallagnol A.M., Zalewski A. (2024). Chemical Composition of Volatile and Extractive Components of Canary (Tenerife) Propolis. Molecules.

[32] Houten S.M., Denis S., Argmann C.A., Jia Y., Ferdinandusse S., Reddy J.K., Wanders R.J.A. (2012). Peroxisomal L-Bifunctional Enzyme (EHHADH) Is Essential for the Production of Medium-Chain Dicarboxylic Acids. J. Lipid Res..

[33] Chen H., Cheng X., Pan X., Yao Y., Chen L., Fu Y., Pan X. (2025). Metabolomic Profiling Uncovers Diagnostic Biomarkers and Dysregulated Pathways in Parkinson’s Disease. Front. Neurol..

[34] Amssayef A., Bouadid I., Eddouks M. (2023). L-Tartaric Acid Exhibits Antihypertensive and Vasorelaxant Effects: The Possible Role of eNOS/NO/cGMP Pathways. Cardiovasc. Hematol. Agents Med. Chem..

[35] Pei Y., He Y., Wang X., Xie C., Li L., Sun Q., Liu L., Shan S., Wang P., Liu T. (2024). Tartaric Acid Ameliorates Experimental Non-Alcoholic Fatty Liver Disease by Activating the AMP-Activated Protein Kinase Signaling Pathway. Eur. J. Pharmacol..

[36] Reith M.E.A., Kramer H.K., Sershen H., Lajtha A. (1990). D-Tartrate Alters Uptake of [^3^H]Dopamine into Brain Synaptic Vesicles. J. Neurosci. Methods.

[37] Nigam S.K., Bush K.T., Martovetsky G., Ahn S.-Y., Liu H.C., Richard E., Bhatnagar V., Wu W. (2015). The Organic Anion Transporter (OAT) Family: A Systems Biology Perspective. Physiol. Rev..

[38] Goes A.T.R., Jesse C.R., Antunes M.S., Lobo Ladd F.V., Lobo Ladd A.A.B., Luchese C., Paroul N., Boeira S.P. (2018). Protective Role of Chrysin on 6-Hydroxydopamine-Induced Neurodegeneration in a Mouse Model of Parkinson’s Disease: Involvement of Neuroinflammation and Neurotrophins. Chem.-Biol. Interact..

[39] Del Fabbro L., Rossito Goes A., Jesse C.R., de Gomes M.G., Cattelan Souza L., Lobo Ladd F.V., Lobo Ladd A.A.B., Nunes Arantes R.V., Reis Simionato A., Oliveira M.S. (2019). Chrysin Protects against Behavioral, Cognitive and Neurochemical Alterations in a 6-Hydroxydopamine Model of Parkinson’s Disease. Neurosci. Lett..

[40] Chen Q.X., Zhou L., Long T., Qin D.L., Wang Y.L., Ye Y., Zhou X.G., Wu J.M., Wu A.G. (2022). Galangin Exhibits Neuroprotective Effects in 6-OHDA-Induced Models of Parkinson’s Disease via the Nrf2/Keap1 Pathway. Pharmaceuticals.

[41] Berretta A.A., Nascimento A.P., Bueno P.C.P., de Oliveira Lima Leite Vaz M.M., Marchetti J.M. (2012). Propolis Standardized Extract (EPP-AF^®^), an Innovative Chemically and Biologically Reproducible Pharmaceutical Compound for Treating Wounds. Int. J. Biol. Sci..

[42] Silveira M.A.D., De Jong D., Berretta A.A., Galvão E.B.d.S., Ribeiro J.C., Cerqueira-Silva T., Amorim T.C., Conceição L.F.M.R.d., Gomes M.M.D., Teixeira M.B. (2021). Efficacy of Brazilian Green Propolis (EPP-AF^®^) as an Adjunct Treatment for Hospitalized COVID-19 Patients: A Randomized, Controlled Clinical Trial. Biomed. Pharmacother..

[43] Silveira M.A.D., Menezes M.d.A., de Souza S.P., Galvão E.B.D.S., Berretta A.A., Caldas J., Teixeira M.B., Gomes M.M.D., Damiani L.P., Bahiense B.A. (2023). Standardized Brazilian Green Propolis Extract (EPP-AF^®^) in COVID-19 Outcomes: A Randomized Double-Blind Placebo-Controlled Trial. Sci. Rep..

[44] Silveira M.A.D., Teles F., Berretta A.A., Sanches T.R., Rodrigues C.E., Seguro A.C., Andrade L. (2019). Effects of Brazilian Green Propolis on Proteinuria and Renal Function in Patients with Chronic Kidney Disease: A Randomized, Double-Blind, Placebo-Controlled Trial. BMC Nephrol..

[45] Paxinos G., Watson C. (1998). The Rat Brain in Stereotaxic Coordinates.

[46] Garcia A., Barbas C., Metz T.O. (2011). Gas Chromatography–Mass Spectrometry (GC-MS)-Based Metabolomics. Metabolic Profiling.

[47] Mastrangelo A., Ferrarini A., Rey-Stolle F., García A., Barbas C. (2015). From Sample Treatment to Biomarker Discovery: A Tutorial for Untargeted Metabolomics Based on GC-(EI)-Q-MS. Anal. Chim. Acta.

[48] Vallejo M., García A., Tuñón J., García-Martínez D., Angulo S., Martin-Ventura J.L., Blanco-Colio L.M., Almeida P., Egido J., Barbas C. (2009). Plasma Fingerprinting with GC-MS in Acute Coronary Syndrome. Anal. Bioanal. Chem..

