# Supplementary material for: Striatal Metabolomic Profiling Links Brazilian Green Propolis to Suberic Acid Modulation and Nigrostriatal Neuroprotection in a Rat Model of Parkinson’s Disease
Source: Molecules. 2026 May 23;31(11):1791. doi: 10.3390/molecules31111791 (PMC13257768; doi:10.3390/molecules31111791)
Supplement: Supplementary file 1 [file molecules-31-01791-s001.zip › molecules-4149277-supplementary.pdf]

Supplementary Materials

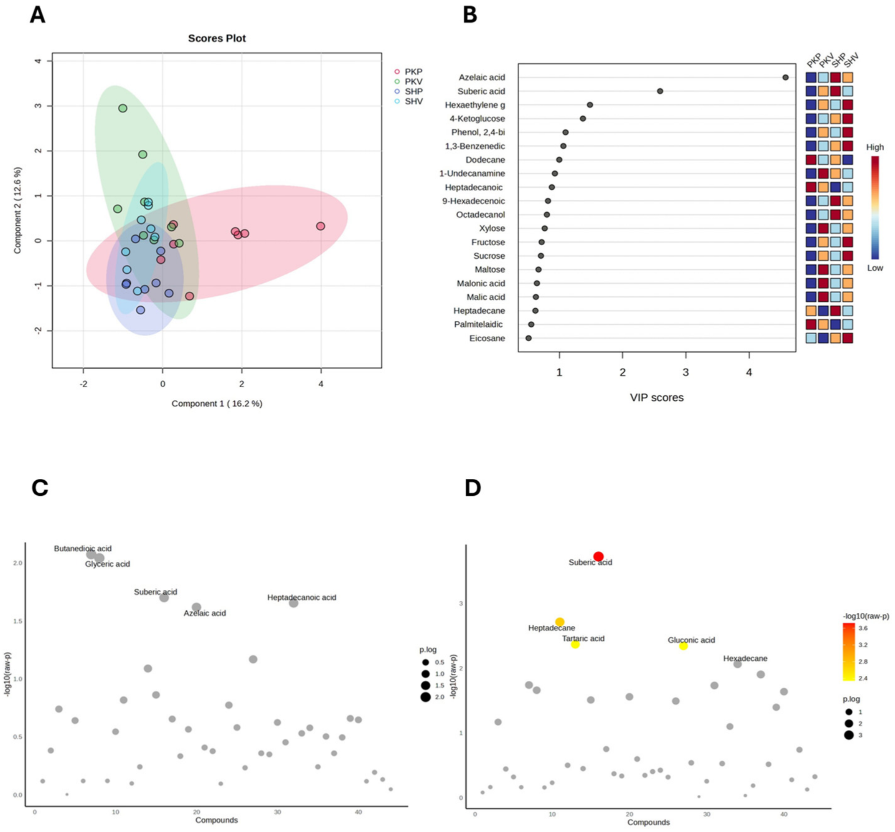

Figure S1. Multivariate and univariate metabolomic analyses before and after outlier removal. (A) PLS-DA score plot showing the distribution of samples among experimental groups (PKP, PKV, SHP, and SHV) including the detected outlier. The percentages on the axes represent the variance explained by Component 1 (16.2%) and Component 2 (12.6%). (B) Variable Importance in Projection (VIP) scores identifying the metabolites that most contributed to group discrimination; the heatmap indicates the relative abundance of these metabolites across groups (red = higher abundance, blue = lower abundance). (C) Univariate analysis including the outlier, displaying metabolites according to −log10(raw p-value), with the most relevant compounds labeled. (D) Univariate analysis after outlier removal, highlighting the metabolites that remained significantly associated with group differences after exclusion of the outlier.

Table S1. Metabolites detected in untargeted GC–MS metabolomic analysis of striatal tissue across the experimental groups. Compounds are listed according to their chromatographic retention index (Ret. Index).

Compound Name	Ret. Index	QA (%)	F-stat	p-value	FDR	Tukey's HSD
Suberic acid	1075	13.616	10.862	0.000189	0.008337	PKV–PKP, SHP–PKP, SHV–PKP
Heptadecane	1711	4.530	7.1186	0.001937	0.042613	PKV–PKP, SHP–PKV
Tartaric acid	1642	6.729	6.0227	0.004281	0.0498	PKV–PKP, SHP–PKP, SHV–PKP
Gluconic acid	2157	3.945	5.9485	0.004527	0.0498	SHV–PKP, SHV–PKV
Hexadecane	1753	5.000	5.1207	0.008628	0.075928	
2-Methyltetracosane	2442	1.790	4.6679	0.01249	0.091592	
Butanedioic acid	1170	3.348	4.2243	0.018174	0.10126	
Tetracosane	2407	2.619	4.2043	0.018488	0.10126	
Glyceric acid	1199	0.704	4.0101	0.021883	0.10126	
1,3-Benzenedicarboxylic acid	2704	5.494	3.9526	0.023013	0.10126	
Azelaic acid	1667	15.406	3.7465	0.027622	0.10881	
Ribonolactone	1698	15.996	3.6212	0.030907	0.10881	
Eicosane	2009	1.685	3.5776	0.032148	0.10881	
Maltose	2846	17.789	3.3361	0.04008	0.12597	
Dodecane	1214	19.655	2.7787	0.067753	0.19874	
Octadecanol	2153	3.425	2.6139	0.079478	0.21856	
1-Undecanamine	1363	5.415	1.8139	0.1771	0.44359	

Phenol, 2,4-bis(1,1-dimethylethyl)	4356	7.061	1.7902	0.18147	0.44359	
Arabinaric acid	1929	8.984	1.4725	0.25218	0.584	
Palmitelaidic acid	1995	12.737	1.3436	0.28851	0.60261	
Heptadecanoic acid	2087	8.196	1.3177	0.29644	0.60261	
Sucrose	3552	6.390	1.2938	0.30391	0.60261	
Threonic acid	1563	1.707	1.2596	0.315	0.60261	
Xylose	1664	20.141	1.1461	0.35476	0.63297	
Urea	1136	10.340	1.133	0.35964	0.63297	
Glucose	1923	1.336	1.0925	0.37519	0.63494	
Tagatose	1889	2.947	1.0453	0.39419	0.63976	
Ribonic acid	1787	2.126	0.97257	0.42523	0.63976	
Fructose	1912	12.295	0.91778	0.45016	0.63976	
Tricine	1959	24.709	0.89484	0.461	0.63976	
9-Hexadecenoic acid, eicosyl ester	3775	17.280	0.87092	0.47256	0.63976	
Phosphoric acid	1276	6.632	0.86226	0.47681	0.63976	
Allose	1937	2.582	0.85617	0.47982	0.63976	
Monostearin	2780	9.353	0.76391	0.52757	0.68273	
Palmitic acid	2044	12.241	0.71246	0.55597	0.69893	
Malic acid	1486	17.537	0.66037	0.58602	0.71625	
Hexaethylene glycol	2162	14.831	0.55839	0.64859	0.76284	
Malonic acid	1202	29.414	0.50373	0.68405	0.76284	
Glycine	1316	19.585	0.49905	0.68715	0.76284	
Tartronic acid	1290	24.761	0.4895	0.69349	0.76284	
9-Hexadecenoic acid, octadecyl ester	3576	21.933	0.41463	0.74435	0.79881	

Glycolic acid	1075	4.267	0.29475	0.82872	0.86818	
Stearic acid	2186	10.147	0.15675	0.9241	0.94559	
Isopropyl palmitate	2013	8.935	0.098848	0.95973	0.95973	

Compound identification was performed based on spectral matching with reference libraries, and the quality of identification is represented by the matching score (QA, %). Statistical differences among groups were evaluated using one-way ANOVA (F-statistic and p-value), followed by false discovery rate correction (FDR, Benjamini–Hochberg method). Pairwise group comparisons were assessed using Tukey's honestly significant difference (HSD) post hoc test. Significant pairwise contrasts are indicated in the Tukey's HSD column. Experimental groups were defined as SHV (Sham + Vehicle), SHP (Sham + Propolis), PKV (Parkinson + Vehicle), and PKP (Parkinson + Propolis).

Table S2. Quality control (QC) injections used to evaluate the analytical stability and reproducibility of the GC–MS platform during the metabolomic analysis.

Injection	Signal Intensity
QC4_1	1202586
QC5_1	1323644
QC6_1	1423590
QC7_2	1481261
QC8_2	1498521
QC9_2	1596005

CV (%): 8.77 Tolerance (%): 30
